# The Expanded Evidence-Centered Design (e-ECD) for Learning and Assessment Systems: A Framework for Incorporating Learning Goals and Processes Within Assessment Design

**DOI:** 10.3389/fpsyg.2019.00853

**Published:** 2019-04-26

**Authors:** Meirav Arieli-Attali, Sue Ward, Jay Thomas, Benjamin Deonovic, Alina A. von Davier

**Affiliations:** ^1^ ACTNext, ACT Inc., Iowa City, IA, United States; ^2^ Fordham University, New York City, NY, United States; ^3^ ACT Inc., Iowa City, IA, United States

**Keywords:** task design, technology-based assessment, blended assessment and learning, development framework, Evidence model

## Abstract

Evidence-centered design (ECD) is a framework for the design and development of assessments that ensures consideration and collection of validity evidence from the onset of the test design. Blending learning and assessment requires integrating aspects of learning at the same level of rigor as aspects of testing. In this paper, we describe an expansion to the ECD framework (termed e-ECD) such that it includes the specifications of the relevant aspects of learning at each of the three core models in the ECD, as well as making room for specifying the relationship between learning and assessment within the system. The framework proposed here does not assume a specific learning theory or particular learning goals, rather it allows for their inclusion within an assessment framework, such that they can be articulated by researchers or assessment developers that wish to focus on learning.

## Introduction

There is a growing need for the development of assessments that are connected and relevant to learning and teaching, and several attempts have been made in recent years to focus on this topic in conferences and journals. For example, Mark Wilson’s 2016 June and September presidential messages in the National Council for Measurement in Education’s newsletter addressed Classroom Assessment, and this topic was also the conference theme for the following 2 years, 2017 and 2018. The journal *Assessment in Education: Principles, Policy & Practice* recently devoted a special issue on the link between assessment and learning (volume 24, issue 3, 2017). The issue focused on the developments in the two disciplines which, despite mutual influences, have taken distinctly separate paths over time. In recent years, systems that blend learning and assessment have been proposed all over the world (e.g., Razzaq et al., 2005; Shute et al., 2008; Feng et al., 2009b; Attali and Arieli-Attali, 2014; Straatemeier, 2014). While within the educational measurement field, there are established standards and frameworks for the development of reliable and valid assessments, those rarely take learning aspects into account. As part of our own effort to develop a blended learning and assessment system, we identified a need for a formal framework of development that includes aspects of learning at the same level of detail and rigor as aspects of testing. This paper describes our general approach at expanding an assessment framework, with some examples from our system to better illustrate the abstract concepts.

Our approach at expanding a principled assessment design is primarily concerned with the inclusion of three dimensions: *aspects of learning*, such as the ability to incorporate the change over time in the skills to be measured at the conceptual level; *aspects of interactive and digital instructional content*, such as simulations, games, practice items, feedback, scaffolds, videos, and their associated affordances for the data collection in rich logfiles; and *measurement models for learning* that synthesize the complexities of the digital instruction and data features.

The expanded framework proposed here allows for the design of systems for learning that are principled, valid, and focused on the learner. Systems designed in this framework are intrinsically connected with the assessment of the skills over the time of instruction, as well as at the end, as summative tests, if so desired. This type of systems has an embedded efficacy structure, so that additional tests can be incorporated within. Learning and assessment developers, as well as researchers, can benefit from such a framework, as it requires articulating both the assessment and learning intended goals at the start of the development process, and it then guides the process to ensure validity of the end-product. The framework proposed here does not assume a specific learning theory or particular learning goals, rather it allows for their inclusion within the assessment framework. The measurement perspective, combined with the learning sciences perspective in the development of content, provides a new and significant shift in the modern development of leaning and assessment systems.

**Figure 1:**
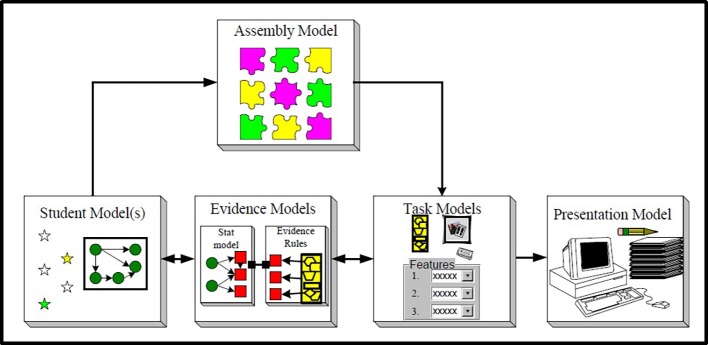
The core models within the ECD framework (from Mislevy Almond & Lucas, © 2003 Educational Testing Service; used with permission); note that later versions term the Student model as Proficiency model.

We chose to expand the well-known evidence-centered design framework (ECD; Mislevy et al., 1999, 2003, 2006). The ECD formulates the process of test development to ensure consideration and collection of validity evidence from the onset of the test design. The ECD is built on the premise that a test is a measurement instrument with which specific claims about the test scores are associated, and that a good test is a good match of the test items and the test takers’ skills. The ECD framework defines several interconnected models, three of which form the core of the framework and are relevant to our discussion: the Student model(s), Evidence model(s), and Task model(s) (the combination of the three models is also called the Conceptual Assessment Framework; CAF; see Figure 1). Note that in more recent publications of the ECD, the Student model is termed a Proficiency model (e.g., Almond et al., 2015).

The Student or the Proficiency model(s) specifies the knowledge, skills, and ability (KSA; which are *latent* competencies) that are the target of the test. This model can be as simple as defining one skill (e.g., the ability θ) or a map of interconnected subskills (e.g., fractions addition, subtractions, multiplication, and division are interconnected subskills that form the map of knowing fractions). The latent competencies that are articulated and defined in this model establish the conceptual basis of the system, and they are often based on a theory or previous findings related to the goal of the assessment.

Since we cannot tap directly into the latent competencies, we need to design tasks/test items such that they will elicit behaviors that can reflect on or indicate about the latent competencies. This is the role of the Task model(s). The Task model specifies the *tasks features* that are supposed to elicit the observables, and only them, such that to allow inferences about the latent competencies. For example, if the assessment is intended to measure “knowledge of operating with fractions,” the tasks should be designed with care such that reading ability is not an obstacle to perform well on the task and express one’s fractions knowledge.

The Evidence models then make the connection between the latent competencies [specified by the Student/Proficiency model(s)] and the observables [behaviors elicited by the Task model(s)]. In other words, the Evidence models are the connecting link. The Evidence models include the measurement model, comprised of the rubrics, the scoring method, and the statistical method for obtaining a total score(s). See Figure 1 for a diagram of the ECD and specifically the three CAF models (note that latent competencies are symbolized as circles, while observables as squares; and the connection between the circles and squares are shown in the Evidence models).

Two important additional models are the Assembly model and the Presentation model (see Figure 1). The Assembly model defines how the three models in the CAF (the Student/Proficiency, Task, and Evidence models) work together and specifically determines the conditions for reliability and validity of the system. As part of the Assembly model, the developers determine the number of items/tasks and their mix (“constraints”) such they provide the necessary evidence and are balanced to properly reflect the breadth and diversity of the domain being assessed. The Presentation models are concerned with different ways to present the assessment, whether it is a paper-and-pencil test, a computer-based test, a hands-on activity, etc. We will elaborate on and delve deeper into each of the models as part of the expansion description below; for more details on the original ECD, see Mislevy et al. (2003, 2006).

There are other alternatives frameworks for the design and development of assessment that follow a principled approach, such as the Cognitive Design System (Embretson, 1998), the Assessment Engineering framework (Luecht, 2013), the Principled Design for Efficacy framework (Nichols et al., 2015), or the Principled Assessment Design framework (Nichols et al., 2016). These frameworks may be perceived as alternatives to the ECD, and one might find any of them as a candidate for a similar expansion the way we demonstrate executing for the ECD in this paper. The reason there were several assessment frameworks developed over the years stem from the need to ensure validity of assessment tools. Although traditional assessments were developed for about half a century without a principled approach (i.e., by following an assessment manual and specifications) and validity was verified after development, the advantage of following a principled framework such as the ECD or others is particularly evident when the goal is to assess *complex competencies* (e.g., problem solving, reasoning, collaborative work) and/or when using *complex performance tasks* (e.g., multidimensional tasks such as performance assessment, simulations or games on computer or otherwise). In these cases, it is important to explicitly identify the relevant competencies and behaviors and how they are connected, because the complexity of the focal competencies and/or the rich data that the tasks provide might pose difficulties in making inferences from behaviors to competencies. ECD has been also successfully applied to address the challenges of simulation- and game-based assessment (Rupp et al., 2010a; Mislevy, 2013; Kim et al., 2016).

## Motivation for a Principled Approach to the Design and Development of a Learning and Assessment System

Learning and assessment, although both relate to the process of determining whether or not a student has a particular knowledge, skill, or ability (KSA), differ substantially in the way they treat KSAs. The main difference between an assessment tool and a learning tool is in the *assumption* about the focal KSA, whether it is fixed or dynamic at the time of interacting with the tool. The Student/Proficiency model in the ECD describes a map of competencies (KSAs), and as in most psychometric models for testing, the assumption is of a latent trait, which is “fixed” at the time of taking the test. The purpose of an assessment is thus to “detect” or “diagnose” that fixed latent KSA at a certain point in time, similar to any measurement tool (e.g., a scale measuring a person’s weight at a particular point in time). On the other hand, the main purpose of a learning tool, such as a computer tutoring system, is to “move” the learner from one state of knowledge to another – that is, the concern is first and foremost with the *change* in KSAs over time, or the *transition*. Of course, an assessment tool *per se* cannot drive the desired change unless deliberate efforts are implemented in the design of the system (similar to a scale which will not help with weight loss unless other actions are taken). Thus, systems that aim at blending assessment and learning cannot implement ECD as is, since ECD is inherently a framework to develop assessments and not learning.

Moreover, the availability of rich data collected *via* technology-enhanced learning and assessment systems (e.g., trial and error as part of the learning process, hint usage) poses challenges, as well as promises, for assessment design and the decision process of which actions to allow and what to record, either to promote efficient learning or to enable the reliable assessment of the learning in order to make valid inferences about KSAs. Computational Psychometrics (von Davier, 2017), an emerging discipline, blends theory-based methods and data-driven algorithms (e.g., data mining and machine learning) for measuring latent KSAs. Computational Psychometrics is a framework for analyzing large and often unstructured data, collected during the learning or performance process, on a theoretical learning and psychometric basis. We also combine aspects of Computational Psychometrics in our expanded design framework, similar to previous accounts that integrated data mining into ECD (e.g., Mislevy et al., 2012; Ventura and Shute, 2013). Combining data-driven algorithms into ECD allows knowledge discovery and models’ update from data, thereby informing the theory-based Student/Proficiency model and enriching the Evidence model.

Attempts to develop innovative assessments within games or as part of complex skills assessment and learning also brought about variations or expansions to ECD (e.g., Feng et al., 2009a; Conrad et al., 2014; Grover et al., 2017). One characteristic of ECD variants focuses on the task and its connection to the Evidence model. Since game-play and the rich data from complex assessments often result in sequences of actions, not all of which are relevant to the target competencies, researchers may follow an ECD approach with expansion with respect to the action-data, to specify which actions are relevant and should be included in the Evidence model and in what way (i.e., expansion on the scoring rules or both scoring and Task model). Such an attempt was done by Grover et al. (2017). Grover and her colleagues expanded on the scoring rules by employing data driven techniques (e.g., clustering, pattern recognition) in addition to theory-based hypotheses, to guide the definition of the scoring rules. Another interesting variation is the experiment-centered design by Conrad et al. (2014), which illustrated an expansion on the scoring and the Task model. This approach uses an ECD-like process to simultaneously encode actions of players in one way for game design and another way for assessment design. Because the game design dictates feedback on actions, and subsequent game options may depend on student’s actions, the game designer needs to encode the actions differently than a researcher or an assessment designer, who is primarily interested in estimating whether a student possesses the focal skill. In this procedure, the model is first postulated around the task (experiment), and then applied separately as two models (versions), one for the game designer, and one for the researcher, each focused on a different encoding of student actions. However, there is only one Evidence model for inferring KSAs, derived from the researcher’s version of the task encoding (the assessment variant scoring rule). In this way, the adaptation of the ECD allowed adding the assessment as a “layer” on top of the game design (stealth assessment), while ensuring coordination between these two layers.

Work by Feng et al. (2009a) is particularly relevant in this context. The authors examine an adaptation of the ECD for learning data (ECDL), applied retroactively to the ASSISTments data (Heffernan and Heffernan, 2014). The ECDL is an ECD with an augmented *pedagogical model*, which has links to all three models of the CAF (Proficiency, Evidence, and Task). The pedagogical model refers to the learning and learners’ characteristics, including learning effectiveness and efficiency (e.g., reducing cognitive load, increasing difficulty gradually during presentation, adapting the presentation of content, and decomposing multistep problems to sub-steps), as well as learner engagement factors. Since ASSISTments was initially developed without ECD in mind, the analysis retroactively checks which claims can support a validity argument that an item with its hints and scaffolds serves the learning goal. This is done by identifying (within each item) the KSAs required to answer it correctly, tagging each as “focal” or “unfocal.” The focal KSAs are the ones which the hints/scaffolds should address. The relation between the focal and unfocal also serves as an indication of the system’s efficacy [a system with a high proportion of unfocal KSAs is less efficient than a system with a low proportion, because this reflects the proportion of KSAs not taught (scaffolded)]. In sum, Feng and his colleagues demonstrated how an existing learning product can be analyzed (and potentially improved) using an ECDL framework.

Common to the various adaptations of ECD is that they were task driven. First came the tasks; then came the ECD analysis, which resulted in adapting the ECD to address the complexity and intuition that were built into the tasks, expressed as an expansion on one of the three models in the CAF. While in the first two examples of Conrad et al. (2014) and Grover et al. (2017), the revised ECD focused on how to encode the task data to feed into the Evidence model, Feng et al.’s (2009a) study goes further, suggesting a pedagogical model that is feeding and being fed by all three CAF models – Proficiency, Evidence, and Task. However, this pedagogical model seems somewhat like a “black box” that retroactively includes the intuitions that specified the product design (e.g., how hints and scaffolds were determined). Additionally, it neither specifies the nature of the connections with the original ECD models nor does it inform how to design a learning product from scratch (i.e., a principled approach to development).

We offer a comprehensive expansion of the ECD framework, such that learning aspects are specified for each of the three models in the CAF and are determined *a priori* to the system design. We describe the expanded full CAF first, followed by a focus on each expanded model with examples. We then discuss the Assembly model, which allows for the specification of the relationship between assessment and learning. We conclude with ramifications of the expanded framework for the development of *adaptive* systems. We include examples to better illustrate the general ideas, along with directions for alternative decisions, to emphasis the generalizability of the expanded framework.

## The Expanded ECD Model

**Figure 2:**
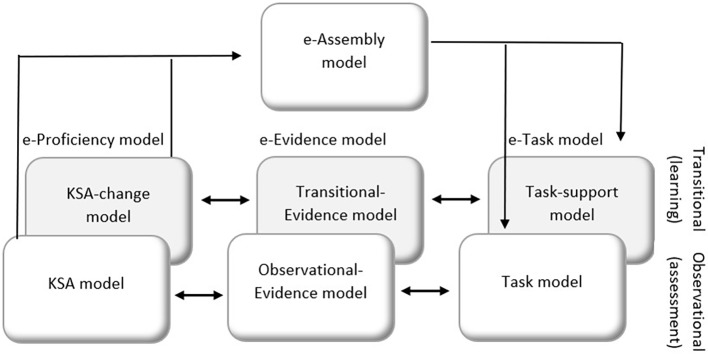
Expanded ECD (e-ECD) for learning and assessment systems.

In our expanded ECD framework (e-ECD), we find it necessary to expand on all three Student/Proficiency, Evidence, and Task models. We do so by adding a *learning layer*, in parallel to the assessment layer. This learning layer can be viewed as a breakdown of a pedagogical model (Feng et al., 2009a) to three components, the conceptual (student/proficiency), behavioral (task), and statistical (evidence) components. Thus, each original ECD model now has an additional paired learning model, culminating in six models. We call each assessment-learning pair an expanded model (e-model), i.e., the e-Proficiency model, the e-Task model, and the e-Evidence model (see Figure 2). Note that we refer to the original Proficiency model as the KSA model (Knowledge, Skills, and Ability), which is now part of the e-Proficiency model.

Within each e-model, we denote an “observational” layer for the assessment aspect (these are the original ECD models with slight title change; the KSA model, Task model, and Observational-Evidence model) and a “transitional” layer for the learning aspect (these are the new models that address learning). The three new learning models include the following: (1) at the conceptual latent level and part of the e-Proficiency model – the transitional layer specifies *learning processes* as the latent competency that the system targets. We denote it as the KSA-change model; (2) at the behavioral level and part of the e-Task model – the transitional layer specifies principles and features of *learning support* that guides the design of tasks (customized feedback, scaffolds, hints, solved examples, solution, or guidance to digital instructional content such as animation, simulation, games, and videos). We denote it as the Task-support model; and (3) at the statistical level and part of the e-Evidence model – the transitional layer specifies the links between the learner’s support usage and the target learning processes, to allow inferring from behaviors to latent learning (e.g., the efficiency of the support used in achieving learning). The data could be large process data and may reveal behavior patterns that were not identified by the human expert in the original e-Proficiency model. In this framework, the e-Proficiency model and the e-Evidence model are supposed to “learn” in real time (be updated) with the new knowledge inferred from the data. We denote it as the Transitional-Evidence model.

We include also an expansion on the Assembly model, denoted e-Assembly model. In addition to determining the number and mix of tasks, the e-Assembly model also includes the specification about the relationship between the assessment component and the learning component of the system and determines how they all work together. In other words, the assembly model determines the “structure” of the system, e.g., when and how learning materials appear and when and how assessment materials appear, and the rules for switching between the two.

Consider the following situation: a student is using a system for learning and assessment to learn and practice scientific reasoning skills. At some point, the student gets an item wrong. In a regular assessment system, another item will follow (often without any feedback about the correctness of the response) – and if the system is an adaptive testing system, the student will receive an easier item, but not necessarily with the same content as the item with the incorrect response. In a blended learning and assessment system, the approach is different. *Detecting a “weakness” in knowledge is a trigger to foster learning*. How should the system aim at facilitating learning? There are several different options, from providing customized feedback and hints on how to answer that specific item, presenting scaffolds for the steps required or eliciting prior knowledge that is needed to answer that item, addressing specific misconceptions that are known to be prevalent for that specific node of KSA, up to re-teaching the topic and showing worked examples, and/or presenting similar items to practice the skill. In many learning products today, this process of defining the learning options is conducted using content experts according to implicit or explicit learning goals. Using a principled approach to development will dictate that the definition of the options for learning should be *explicitly* articulated at the level of the Task-support model, and these features are to be in line with the explicit conceptual learning/pedagogical model that describes how to make that shift in knowledge, i.e., the KSA-change model. The links between the supports and the conceptual KSA-change are defined in the Transitional-Evidence model *via* statistical models, which provide the validity learning argument for the system.

In the development of an assessment system that blends learning, we wish to help students learn, and to validate the claim that learning occurred, or that the system indeed helped with the learning *as intended*. The KSA-change specifies the type of changes (learning/transitions) the system is targeting, and based on that, the tasks and the task supports are defined. In other words, the first step is to define the “learning shifts” or how to “move” in the KSA model from one level/node to the next. The next step is to define the observables that need to be elicited and the connections between the learning shifts and the observables. We elaborate on each of the expanded models below.

Our expanded framework shows how to incorporate a learning theory or learning principles into the ECD and can be applied using different learning approaches. We illustrate this process by using examples from Knowledge-Learning-Instruction (Koedinger et al., 2012) among others, but this process can be applied using other learning approaches (and we provide some directions).

### Expanded Proficiency Model

In the ECD framework, the Student/Proficiency model defines the Knowledge, Skills, and Ability (KSA) that the assessment is targeting. Although in early publications of the ECD, it is called a Student model, in recent contexts, it is called a “Proficiency model” (e.g., Feng et al., 2009a; Almond et al., 2015), or referred to as a “Competency model” (e.g., Arieli-Attali and Cayton-Hodges, 2014; Kim et al., 2016), and it can also be perceived as a “Construct map” (Wilson, 2009). A similar notion in the field of Intelligence Tutoring Systems is a “Domain model” (Quintana et al., 2000), a “Knowledge model” (Koedinger et al., 2012; Pelánek, 2017), or a “Cognitive model” (Anderson et al., 1995). In the Intelligence Tutoring Systems’ literature, the term “Student model” is reserved to a specific map of skills as estimated for a *particular student* – which is an overlay on the domain model (aka the expert model). Within ECD, the Student/Proficiency model includes both the desired skills (that an expert would possess) and the updated level of skills for each particular student following responses on assessment items. To avoid confusion, within our expanded ECD, we refer to it by the general name of a KSA model.

The KSAs are assumed to be latent, and the goal of the assessment is to infer about them from examinee’s responses to test items. When the assessment tool is also intended to facilitate learning (i.e., the system provides supports when the student does not know the correct answer), the assumption is that the student’s level of KSA is *changing* (presumably becoming higher as a result of learning). In the e-ECD, we define a “KSA-change model” that together with the original KSA model creates the expanded-Proficiency model (e-Proficiency model). The KSA-change model specifies the latent *learning processes* that need to occur in order to achieve specific nodes in the KSA model. Each node in the KSA model should have a corresponding *learning-model* in the KSA-change model, which may include prerequisite knowledge and misconceptions, and/or a progression of skills leading up to that KSA node, with the pedagogical knowledge of how to make the required knowledge-shift. Some examples of learning models are learning progressions (National Research Council (NRC), 2007; e.g., Arieli-Attali et al., 2012) a Dynamic Learning Map (Kingston et al., 2017), or learning models based on the body of work on Pedagogical Content Knowledge (Posner et al., 1982; Koehler and Mishra, 2009; Furtak et al., 2012). The importance of Pedagogical Content Knowledge is in considering the interactions of *content information*, *pedagogy*, and *learning theory*. Another approach from the learning sciences and artificial intelligence is the Knowledge-Learning-Instruction framework (KLI; Koedinger et al., 2012), which provides a taxonomy to connect knowledge components, learning processes, and teaching options. We will illustrate our KSA-change model specification using the KLI framework, but we will define the e-Proficiency model in a general way such that any other learning theory can be applied instead.

Specifying and explicitly articulating the latent learning processes and progressions that are the target of the learning is a crucial step, since this is what will guide the specification of both the e-Task model and the e-Evidence model. In the following sections, we elaborate and illustrate the KSA and KSA-change models that constitute the e-Proficiency Model.

#### The Assessment Layer of the e-Proficiency Model – The KSA Model

A KSA model includes *variables* that are the features or attributes of competence that the assessment is targeting. The number of variables and their grain size are determined by the potential use of the assessment, and it can range from 1 (e.g., the θ in college admission tests such as the GRE, SAT, and ACT) to several subskills arranged in a map or a net (e.g., a net example, see Mislevy et al., 1999; a math competency map, see Arieli-Attali and Cayton-Hodges, 2014; two versions of a game-based physics competency model, see Kim et al., 2016). These variables can be derived by conducting a *cognitive task analysis* of the skill by experts, analyzing the content domain, or relying on a theory of knowledge and research findings. The variables and their interconnections create a map in which each variable is a *node* connected by a *link* with other nodes (variables). Following analysis of data from student responses (and using the statistical models), values on these variables define the level of mastery or the probability that a particular student possess those particular sub-skills (nodes), i.e., a value will be attached to each node.

**Figure 3:**
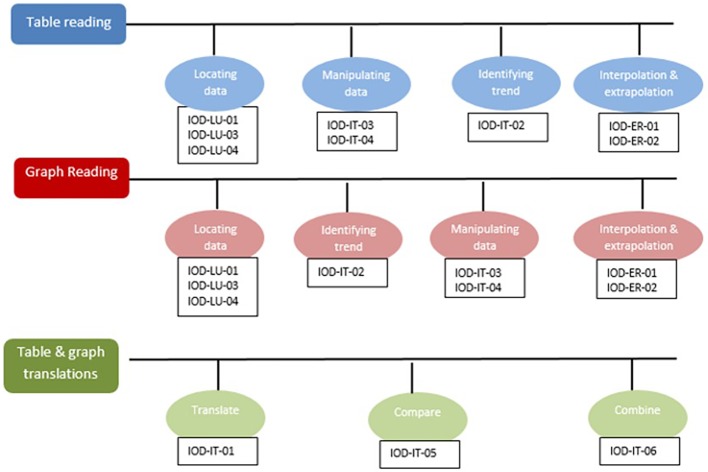
The KSA model for the HERA system for scientific reasoning skills.

As part of our development of a learning and assessment system, called the Holistic Educational Resources & Assessment (HERA) system for scientific thinking skills, we developed a KSA model for *data interpretation* skill. Figure 3 depicts part of the model. Specifically, we distinguish three main skills of data interpretation depending on the data representation (*Table Reading*, *Graph Reading*, and the skill of interpreting data from *both tables and graphs*), and each skill is then divided to several subskills. For example, in *Table Reading* skill, we distinguish between *locating data points*, *manipulating data*, *identifying trend*, and *interpolation and extrapolation*. Note that these same subskills (albeit in a different order) appear also under *Graph Reading* skill, but they entail different cognitive ability. The skill of *Tables and Graphs* includes *comparing*, *combining*, and *translating* information from two or more different representations.

Although KSA models often specify the links between nodes, and may even order the skills in a semi-progression (from basic to more sophisticated skills) as in the example of the HERA model in Figure 3, a knowledge model often does not specify *how to move* from one node to the next, nor does it explicitly define learning processes. To that end we add the learning layer in the e-Proficiency model – the KSA-change model.

#### The Learning Layer in the e-Proficiency Model – The KSA-Change Model

Defining a learning layer within the e-Proficiency model makes room for explicit articulation of the learning processes targeted by the learning and assessment system. The idea is for these specifications to be the result of purposeful planning, rather than a coincidental outcome of system creation. In the Intelligence Tutoring literature, developers consider what they call the “Learner model” (Pelánek, 2017) or the “Educational model” (Quintana et al., 2000) or more generally, processes for knowledge acquisition (Koedinger et al., 2012). This model can also be viewed as the “pedagogical model” and apply principles of Pedagogical Content Knowledge (Koehler and Mishra, 2009; Furtak et al., 2012). We call this model the “KSA-change Model” for generalizability and to keep the connection with the original KSA model, with the emphasis on the *change* in KSA. Using the title “change” makes room also for negative change (aka “forgetting”), which albeit not desirable, is possible.

A KSA-change model is the place to incorporate the specific learning theory or learning principles (or goals) that are at the basis of the systems. Similar to the way a KSA map is created, the KSA-change map should specify the learning aspects of the particular skills. Here we provide a general outline for how to specify a KSA-change model, but in each system this process may take a different shape.

A KSA-change model may include variables of two types:

1. Sequences of knowledge components, features or attributes
2. Learning processes within each sequence

These two types of variables define the learning *sequences* and *processes* that are needed to facilitate learning. The KSA-change variables are derived directly from the KSA model, such that each node/skill in the KSA model has a reference in the KSA-change model in the form of how to “move” students to learn that skill.

**Figure 4:**
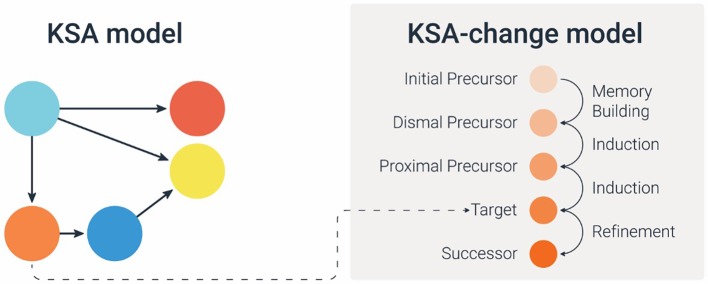
A general diagram of the e-Proficiency model (the orange node in the KSA model is specified in the KSA-change model for learning sequence and learning processes). Similarly, we can construct a sequence for each of the other nodes (the blue, pink, and red nodes).

Given a specific skill (node in the map), this may be done in two stages: (1) the first step is to define the (linear) *sequence* of pre-requisites or precursors needed to learn that target skill (node). For example, Kingston and his colleagues (Kingston et al., 2017) developed Dynamic Learning Maps in which each of the target competencies are preceded with three levels of precursor pieces of knowledge (initial precursor, distal precursor, and proximal precursor) and succeeded by a successor piece of knowledge, together creating what they called “Linkage levels.” When defining the sequence of precursors attention should be given to the grain size, as well as to specific features or attributes of these precursors. In KLI terminology (Koedinger et al., 2012), this would mean to characterize the *Knowledge Components* of the subskills. Some Knowledge Components are: fact, association, category, concept, rule, principle, plan, schema, model, production; and whether it is verbal or non-verbal, declarative or procedural; or integrative knowledge (2) the second step is to characterize the learning sequence by which kind of learning *process* is required to achieve the learning. For example, applying the KLI taxonomy (Koedinger et al., 2012), we can assign to each precursor (knowledge component) a specific learning process that is presumed to make the desired knowledge shift. The KLI framework characterizes three kinds of learning processes: *memory and fluency building*, *induction and refinement*, and *understanding and sense-making*. Specifying which kind of process is needed in the particular learning sequence is necessary for subsequent decisions about the supports to be provided. For example, if the focal learning process is *fluency building*, this implies that the learning system should provide practice opportunities for that KSA. In contrast, if the focal learning process for a different KSA is *understanding and sense making*, then the learning system should provide explanations and examples. Figure 4 illustrates a general e-Proficiency model with an artificial example of adding-on the learning processes to a knowledge sequence built off of three prerequisites and a successor piece.

**Figure 5:**
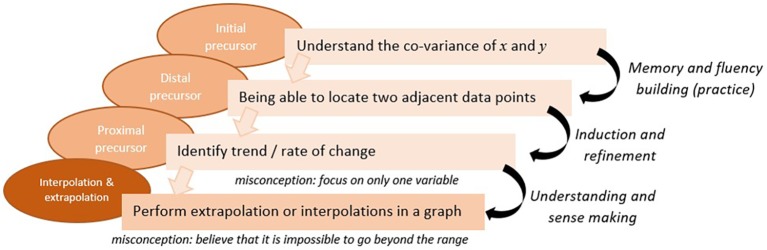
A specification diagram of the KSA-change model for one node/skill of interpolation/extrapolation in a graph in the HERA’s KSA-model.

Applying the above approach to the HERA learning and assessment system, let us focus on the subskill of *interpolation and extrapolation from data in a graph* (the last red circle in the progression of *Graph Reading* skill in Figure 3). Based on our guidelines above, the first step would be to determine a sequence of subskills/precursors and to characterize them, and then as a second step to specify the cognitive process(es) that would make the transition from one subskill to the next. Figure 5 presents one section of the KSA-change of the HERA system for the subskill of *interpolation and extrapolation in a graph*. The model specifies the proximal, distal, and initial precursors as follows: the proximal precursor = *identifying the rate of change in the dependent variable (y-variable) as the independent variable (x-variable) changes*; distal precursor = *being able to locate the y-value for a certain x-value point on a graph, and find adjacent points and compare the relative values*; initial precursor = *understanding that the two variables in a graph are co-related.* Now applying the KLI knowledge components characterization, the proximal precursor (identifying rate of change) may be characterized as “rule”; the distal precursor (locate points and compare) as “schema”; and the initial precursor (two variables are co-related) as a “concept.”

Next, we determine the cognitive processes that foster the transition from one subskill to the next. For example, given an understanding of the co-variation of *x* and *y* (the initial subskill) students need to practice finding the y-points for different x-points to create the mental schema and build *fluency* with locating points and particularly two adjacent points. However, to “jump” to the next step of identifying the trend and the rate of change requires *induction and refinement* to derive the rule. The last transition from identifying rate of change to perform interpolation & extrapolation requires *sense making and deduction* – deducing from the rule to the new situation. Given the specific learning processes, we can later define which learning supports would be most appropriate (e.g., practice for fluency building, worked example and comparisons for induction, and explanation for sense making and deduction). The model in Figure 5 shows the different learning processes as the transitions (arrows) required between the subskills in the sequence. This is the learning model for the specific skill in focus, and is usually derived based on expert analysis. The model in Figure 5 also specifies particular misconceptions that students often exhibit at each level. Specifying misconceptions may also help determine which feedback and/or learning aid to provide to students. We show in the next section how to define Task and Task-support models based on this example.

There are several decisions that are taken as part of the model specifications. One of them is the grain-size of each precursor. An alternative KSA-change model can be determined with smaller or larger grain size subskills. Another decision is whether to adopt a three-level precursor skill structure, or alternatively focus on only one precursor and the different misconceptions students may have. Researchers and developers are encouraged to try different approaches.

We propose to derive the KSA-change variables by conducting a *learning process analysis* by experts, i.e., an analysis of the pedagogical practices in the content domain or relying on a theory of learning in that domain, similar to the way we illustrated above (by using the KLI taxonomy). This is also parallel to the way a KSA model is derived based on *cognitive task analysis* or domain analysis. The KSA-change model constitutes a *collection* of sequences (and their processes), each addressing one node in the KSA model (as illustrated in Figures 4, 5). This can also be viewed as a two-dimensional map, with the sequences as the second dimension for each node.

Similar to updating the KSA model for a student, here too, following analysis of data from student responses and student behaviors in using the learning supports, values on the KSA-change variables indicate level or probability that a particular student has gone through a particular learning process (or that a particular knowledge shift was due to the learning support used). We will discuss this in more detail in the e-Evidence model section.

### Expanded Task Model

In the original ECD framework, the Task model specifies the features of tasks that are presumed to elicit observables to allow inference on the target KSA. An important distinction introduced in ECD is between a task model design based on a Proficiency model and a task-centered design (Mislevy et al., 1999). While in task-centered design, the primary emphasis is on creating the task with the target of inference defined only implicitly, as the tendency to do well on those tasks, in defining a task model based on a Proficiency (and Evidence) model, we make the connections and possible inferences *explicit from the start*, making the design easier to communicate, easier to modify, and better suited to principled generation of tasks (Mislevy et al., 1999, p. 23). Moreover, basing a task model on Proficiency and Evidence models allows us to consider reliability and validity aspects of task features, and particularly the cognitively or empirically based relevance of the task features. In other words, considerations of item reliability and validity guide the development of items to elicit the target observables and *only them* (minimizing added “noise”). This means that at the development stage of a task, all features of the task should stand to scrutiny regarding relevance to the latent KSA. As mentioned above, if reading ability is not relevant as part of the mathematics KSA, items or tasks that may impede students with lower reading skills should be avoided. Thus, defining a task model based on a Proficiency model resembles the relationship between the latent trait and its manifestation in observable behavior. The more the task relates to the target KSA, the better the inference from the observable to the latent KSA.

For assessment precision purposes per-se, there is no need to provide feedback to students; on the contrary, feedback can be viewed as interference in the process of assessment, and likewise scaffolds and hints introduce noise or interference to a single-point-in-time measurement. However, when the assessment tool is also intended for learning, the goal is to support learners when a weakness was identified, in order to help them gain the “missing” KSA. In the e-ECD we define a “Task-support model” that together with the original Task model creates the expanded-Task model (e-Task model). The Task-support model specifies the learning supports that are necessary and should be provided to learners in order to achieve KSA change. Similar to basing the Task model on the KSA model, the Task-support model is based on the KSA-change model. The supports may include customized feedback, hints and scaffolds, practice options, worked examples, explanations, or guidance to further tailored instruction derived from the latent learning processes specified in the KSA-change model. In other words, the supports are determined according to the focal knowledge *change*. We elaborate and illustrate on Task and Task-support models below.

#### The Assessment Layer Within the e-Task Model – The Task Model

The Task model provides a framework for describing *the situation* in which examinees are given the opportunity to exhibit their KSAs, and includes the specifications of the stimulus *materials*, *conditions* and *affordances*, as well as specifications for the *work product* (Mislevy et al., 1999, p. 19). The characteristics of the tasks are determined by the nature of the behaviors that provide evidence for the KSAs. Constructing a Task model from the latent KSA model involves considering the *cognitive aspect of task behavior*, including specifying the features of the situation, the internal representation of these features, and the connection between these representations and the problem-solving behavior the task targets. In this context, variables that affect task *difficulty* are essential to take into account. In addition, the Task model also includes features of task *management* and *presentation*.

Although the Task model is built off of the Proficiency model (or the KSA model in our notation), multiple Task models are possible in a given assessment, because each Task model may be employed to provide evidence in a different form, use different representational formats, or focus evidence on different aspects of proficiency. Similarly, the same Task model and work product can produce different evidence; i.e., different rules could be applied to the same work product, to allow inferences on different KSAs. Thus, it is necessary to define within each Task model the specific variables to be considered in the evidence rules (i.e., scoring rules; we elaborate on this in the next section).

Consider the abovementioned KSA from the HERA model: “*Perform an extrapolation using data from a graph*.” As part of a scientific reasoning skills assessment, this skill is defined in a network of other skills related to understanding data representations, as seen in Figure 5. One possible Task model can be: “Given a graph with a defined range for the *x*-axis variable [*a,b*] and *y* values corresponding to all *x* values in the range, find the *y*-value for an *x*-value outside the range.” That is, we present the learner with a graph (defined by its *x*- and *y*- axes) and a function or paired coordinates (*x, y*) for a limited domain. The question then asks learners to predict the *y*-value of an *x* point which is outside the domain presented in the graph. Because extrapolation assumes the continuation of the trend based on the relationship between variables, a required characteristic of the question is to include this assumption, explicitly or implicitly *via* the context (e.g. stating other variables do not change, or the same experimental procedure was used for a new value). Articulating the assumption is part of the Task model. Another option for an extrapolation Task model could be: “Given a graph with two levels of the dependent variable, both showing a linear relationship with the x-variable (i.e., same relationship trend) but with different slopes, find the y-value for a third level of the dependent variable.” That is, we present the learner with a graph with two linear relationships (two line-graphs), one for level *a* and one for level *b* (for example, *a, b* are levels of weight of different carts, and the linear relationship is between speed and time). The question then asks learners to predict the *y*-value for level *c* (*c > a, b;* larger weight car) for an *x-* point for which we know the *y*-values of level *a* and *b*; that is, extrapolation beyond the data presented. This Task model is more sophisticated than the first one, due to the complexity of the data representation, and thus is tapping into a higher level of the skill.

Another aspect is the operationalization of the Task model in a particular item. Given a Task model, the question can take the form of a direct non-contextualized (what we may also call a “naked”) question, (e.g., asking about a value of *y* given a specific *x*), or it can be contextualized (or “wrapped”) within the context and terminology of the graph (e.g., “suppose the researcher decided to examine the speed of a new cart that has greater weight, and suppose the trend of the results observed is maintained, what would you expect the new result to be?”). The “naked” and “dressed” versions of the question may involve change in the difficulty of the item; however, this change needs to be examined, to the extent that it is construct- relevant or irrelevant. If it is construct-relevant, then it should be included in the Task model as part of the specifications. Other factors may affect the difficulty as well – the type of graphic (bar-graph, line-graph, multiple lines, scatter plot) and the complexity of the relationships between variables (linear, quadratic, logarithmic, increasing, decreasing, one y-variable or more), the familiarity of the context of the task (whether this is a phenomenon in electricity, projectile motion, genetics, etc.), the complexity of the context (commonly understood, or fraught with misconceptions), the response options (multiple choice, or open-ended), the quality of the graph and its presentation (easy or hard to read, presented on a computer, smartphone or a paper, presented as a static graph or interactive where learners can plot points), etc. These factors and others need to be considered when specifying the Task model, and their relevance to the construct should be clearly articulated.

#### The Learning Layer Within the e-Task Model – The Task-Support Model

Tasks for assessment and tasks for learning differ in the availability of options that support learning. When we design tasks for learning, we need to consider the type of “help” or “teaching” that the task affords, with the same level of rigor that we put into the design of the task itself. The Task-support model thus specifies the learning supports that might be necessary and should be provided to students in order to achieve the desired KSA-change (i.e., increase in KSA). Similar to basing the task model on the KSA model, the Task-support model is based on the KSA-change model.

Making room for the specification of the task support *in connection* to the learning processes/goals (the focal KSA-change) is the innovative core of the proposed e-ECD and its significant contribution to the design of learning and assessment systems. Many learning systems include scaffolds or hints to accompany items and tasks, often determined by content experts or teacher experience and/or practices. These hints and scaffolds help answer the particular item they accompany, and may also provide “teaching,” if transfer occurs to subsequent similar items. However, in the design process of the hints and scaffolds, often no explicit articulation is made regarding the intended effect of hints and scaffolds *beyond* the particular question, or in connection to the general learning goals. Often, the hints or scaffolds are task-specific; a breakdown of the task into smaller steps, thus decreasing the difficulty of the task. This is also reflected in the approach to assigning partial credit for an item that was answered correctly with hints, contributing less to the ability estimate (as evidence of lower ability; e.g., Wang et al., 2010). Specifying a Task-support model per each Task model dictates a *standardization* of the scaffolds and hints (and other supports) provided for a given task. How do we specify task supports connected to the focal KSA-change?

If for example, we define a particular (as part of the KSA-change model) learning model similar to the one depicted in Figure 5, we may provide as a task support a “pointer” to the precursors, in the form of a hint or a scaffold. Thus, the scaffolds are not a breakdown of the question to sub-steps, but rather each scaffold points to one of the precursor pieces of knowledge (initial, distal, or proximal precursor). In addition, since we defined the kind of knowledge change between each precursor, we can provide the corresponding support per each desired change. If the knowledge change is related to memory and fluency-building, we may provide more practice examples instead of the scaffold. Similarly, if the knowledge change is related to understanding and sense-making, we may provide an explanation or reasoning, or ask the student to provide the explanation or reasoning (self-explanation was found to be beneficial in some case, Koedinger et al., 2012). It may very well be the case that similar scaffolds will result from explicating a Task-support model following an e-ECD compared to not doing so, however in following this procedure, the design decisions are explicit and easy to communicate, justify, modify, replicate, and apply in a principled development of scaffolds.

Similarly, other features of task support, such as feedback, visuals, and links to a video or wiki page, can be supported by the articulation of the KSA-change and the connection between the two.

Let us illustrate specifying a Task-support model for the example item from HERA described in the previous section. Recall that the item targeted the latent KSA “*Perform an extrapolation using data from a graph*,” and the task materials included a graph with a specified function, asking students to extrapolate a point beyond the given range (i.e., predict the value of *y* for a new *x-value*). Also, recall Figure 5 that depicts the KSA-change model for this particular subskill. Given the proximal, distal, and initial precursors, we can now specify each scaffold to address each of these three precursor skills. Alternatively, we can decide to address only the closest precursor (the proximal) as a scaffold, and if that does not help with answering the question correctly, then refer the student to “learn” the more basic material (e.g., in a different section of the system, or by presenting items/content that target the initial and distal precursor skills). These decisions depend on the system design (e-Assembly model) and may vary from system to system.

**Figure 6:**
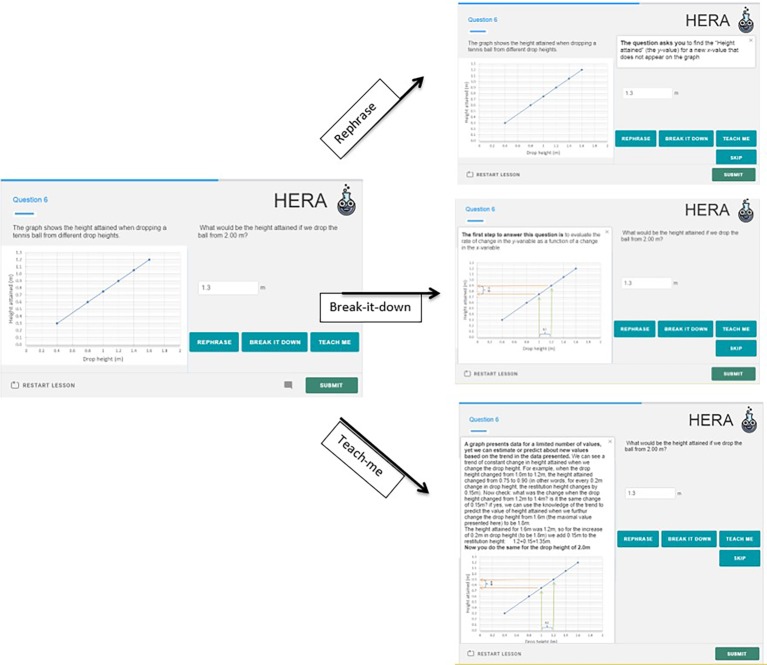
An example of an Assessment & Learning Personalized & Interactive item (AL-PI item) from the HERA system.

As part of our development of the HERA system for scientific thinking skills, we developed an item model that can be used to collect evidence for both assessment and learning, termed an Assessment and Learning Personalized Interactive item (AL-PI). This item looks like a regular assessment item, and only after an incorrect response, the learners are given “learning options” to choose from. We offer three types of learning supports: (1) Rephrase – rewording of the question; (2) Break-it-down – providing the first step out of the multi-steps required to answer the question; and (3) Teach-me – providing a text and/or video explanation of the background of the question. Figure 6 presents a screenshot of an AL-PI item from a task about height-restitution of a dropped-ball, targeting the skill of extrapolation.

Using the terminology above, the Rephrase-option provides the learner with another attempt at the question, with the potential of removing the construct irrelevance that may stem from the item-phrasing (for learners who did not understand what the question is asking them, due to difficulty with the wording). In this example, a Rephrase of the question is: “The question asks you to find the “Height attained” (the *y*-value) for a new *x*-value that does not appear on the graph” (see Figure 6 upper panel). Note that the Rephrase is practically “undressing” (decontextualizing) the question, pointing out the “naked” form, or making the connection between the context and the decontextualized skill.

The second learning support is Break-it-down which takes the form of providing the first step to answer the question. In the example in Figure 6 the Break-it-down states: “The first step to answer this question is to evaluate the rate of change in *y* as a function of a change in the *x*-variable” with additional marks and arrows on the graph to draw the leaner’s attention where to look. The Break-it-down option may look like a hint, signaling to learners where to focus, and in our terminology, it refers to the proximal precursor (recall: proximal precursor = *identifying the rate of the change in the dependent variable as the independent variable changes*).

The third type of support that we offer in an AL-PI item is Teach-me. The Teach-me option in this case includes the following components: (1) a general statement about the skill; i.e., *a graph presents data for a limited number of values, yet we can estimate or predict about new values based on the trend in the data presented*; (2) an explanation of how to identify the trend in a graph, i.e., locating adjacent points; and (3) an illustration of how once the trend was identified, we can perform extrapolation.

In our system we provide an illustration on a different value than the one in the question in order to avoid revealing the correct answer and leaving room for the learner to put mental effort into applying the method taught. In the Task-support model terminology and in relation to the KSA-change model, the Teach-me option addresses all three precursors.

Specifying the task support based on the learning goal and the desired change in KSA gives direction but does not limit the options. On the contrary, it enriches the space of the decision and opens-up new options. In addition, constructing task support by following the e-ECD framework gives rise to the hypothesis that this way of structuring scaffolds may enhance transfer, because the scaffolds do not address the particular question, but rather address the latent skill and its precursor skills. Empirical evidence of transfer is of course needed to examine this hypothesis.

### Expanded Evidence Model

The links made between the e-Proficiency model and the e-Task model need explication of the statistical models that allow inferences from the work products on the tasks to the latent KSAs. In the ECD framework, the Evidence model specifies the links between the task’s observables (e.g., student work product) and the latent KSAs targeted by that task (termed here as Observational-Evidence model). The Observational-Evidence model includes the *evidence rules* (scoring rubrics) and the *statistical models*. The Evidence model is the heart of the ECD, because it provides the “credible argument for how students’ behaviors constitute evidence about targeted aspects of proficiency” (Mislevy et al., 1999, p. 2).

In a system designed for learning, data other than the work product is produced, i.e., the data produced out of the task support (e.g., hints and scaffolds usage), which may be called *process data*. The task support materials are created to foster learning; thus, learning systems should have a *credible argument* that these supports indeed promote learning. Partial evidence for that can be achieved by inferences about knowledge or what students know and can do from their work product in the system, *following* and as *a result of* the use of the supports, and this can be obtained by the statistical models within the Evidence model. However, the efficacy of the task supports themselves (i.e., which support helps the most in which case), and drawing inferences from scaffolds and hint usage about “learning behavior” or “learning processes” (as defined in the KSA-change model) may need new kind of models and evidence. The Transitional-Evidence model within the e-Evidence model addresses the data produced from the task support.

#### The Assessment Layer Within the Evidence Model – The Observational-Evidence Model

In the original ECD, the Observational-Evidence model addresses the question of how to operationalize the conceptual target competencies defined by the Proficiency model, which are essentially latent, in order to be able to validly infer from overt behaviors about those latent competencies. The Observational-Evidence model includes two parts. The first contains the scoring rules, which are ways to extract a “score” or an *observable variable* from student actions. In some cases, the scoring rule is simple, as in a multiple-choice item, in which a score of 1 or 0 is obtained corresponding to a correct or incorrect response. In other cases, the scoring rule might be more complex, as in performance assessment where student responses produce what we call “process data” (i.e., a log file of recorded actions on the task). A scoring rule for process data can take the form of grouping a sequence of actions into a “cluster” that may indicate a desired strategy, or a level on a learning progression that the test is targeting. In such an example, a scoring rule can be defined such that a score of 1 or 0 is assigned corresponding to the respective strategy employed, or the learning progression level achieved. Of course, scoring rules are not confined to dichotomous scores and they can also define scores between 0 and 1, continuous (particularly when the scoring rules relies on response time) or ordered categories of 1-to-*m*, for *m* categories (polytomous scores).

The second part of the Observational-Evidence model contains the statistical model. The statistical model expresses how the scores (as defined by the scoring rules) depend, probabilistically, on the latent competencies (the KSAs). This dependency is probabilistic, that is, the statistical model defines the probability of certain “scores” (observables) given specific latent competencies (combination of values on the KSAs). In other words, at the point in time at which the student is working within the system, that student is in a “latent state” of knowledge, and given that latent state, there is a certain probability for the observable variables, which if observed, are evidence for the latent ability. However, all we have are the student observable variables, and what we need is a psychometric model that allows us to do the *reverse inference* from the given observables to the latent competencies.

There are various statistical models that can be used here. Since we are talking about an assessment and learning system, let us consider a multi-dimensional latent competency, i.e., multiple skills are targeted by the system both for assessment and learning. If we assume the latent competencies to be continuous, we can use a multi-dimensional Item Response Theory models (e.g., MIRT; Reckase, 2009) or Bayes-net models (Pearl, 1988, 2014; Martin and VanLehn, 1995; Chang et al., 2006; Almond et al., 2015). In the case where the latent competencies are treated as categorical with several increasingly categories of proficiency in each (e.g., low-, medium-, and high-level proficiency, or mastery/non-mastery levels), we can use diagnostic classification models (DCM; Rupp et al., 2010b). What these models enable is to “describe” (or model) the relationship between the latent traits and the observables in a probabilistic way, such that the probability of a certain observable, given a certain latent trait, is defined and therefore allow us to make the *reverse* inference – to estimate the probability of a certain level of a latent trait given the observable.

In order to make the link between the items/tasks (the stimuli to collect observables) and the latent KSAs, we can use what is called a Q-matrix (Tatsuoka, 1983). A Q-matrix is a matrix of <items × skills> (items in the rows; skills in the columns), defining for each item which skills it is targeting. The Q-matrix plays a role in the particular psychometric model, to determine the probability of answering an item correctly given the combination of skills (and whether all skills are needed, or some skill can compensate for others; non-compensatory or compensatory model, respectively). The Q-matrix is usually determined by content experts, but it can also be learned from the data (e.g., Liu et al., 2012).

Recent developments in the field of psychometrics have expanded the modeling approach to also include models that are data driven, but informed by theory, and is referred to as Computational Psychometrics (von Davier, 2017). Computational Psychometrics is a framework that includes complex models such as MIRT, Bayes-net and DCM, which allow us to make inferences about latent competencies; however, these models may not define *a priori* the scoring rules, but rather allow for a combination of the expert-based scoring rules with those that are learned from the data. In particular, the supervised algorithms – methodologies used in machine learning (ML) – can be useful for identifying patterns in the complex logfile data. These algorithms classify the patterns by skills using a training data set that contained the correct or theory-based classification. The word supervised here means that the “correct responses” were defined by subject-matter experts and that the classification algorithm learns from these data that were correctly classified to extrapolate to new data points.

In a learning and assessment system, the Observational-Evidence model may also take into account the scaffolds and hints usage to infer about the KSA model. Since the scaffolds and hints reduce the difficulty of the items/tasks, they also change their evidentiary value of the observables. This can be done *via* either using only responses without hint usage to model KSA or applying a partial credit scoring rule for items that were answered correctly with hints, thus assigning them less credit as a reflection of their evidentiary value (e.g., Wang et al., 2010; Bolsinova et al., 2019a,b).

To summarize, any and all statistical models that allow us to define the connection between overt observables and latent competencies can be used in the Observational-Evidence model.

#### The Learning Layer Within the Evidence Model – The Transitional-Evidence Model

Similar to the way the Observational-Evidence model connects the Task model back to the KSA model, the Transitional-Evidence model uses the task supports data to infer about learning, and to link back to the KSA-change model. Recall that the KSA-change model includes pedagogical principles which are reflected in the task supports. Similar to the assessment layer of the Evidence model, the Transitional-Evidence model also includes two parts: the scoring rules and the statistical models.

The scoring rules define the *observable variables* of the Transitional-Evidence model. If task supports are available by choice, student choice behavior can be modeled to make inferences about their learning strategies. The data from the task supports usage (hints, scaffolds, videos, simulations, animations, etc.) as well as number of attempts or response time, should first be coded (according to a scoring or evidence rule) to define which of them should count and in what way. As before, scoring rules can be defined by human experts or can be learned from the data.

**Figure 7:**
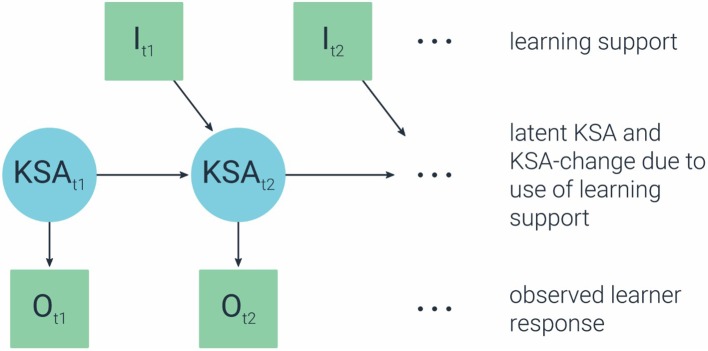
An input-output hidden Markov model (HMM).

The statistical models in the Transitional-Evidence model need to be selected, such that they allow us to infer about *change* based on observables over time. A popular stochastic model for characterizing a changing system is a Markov model (cf. Norris, 1998). In a Markov model, transition to the next state depends only on the current state. Because the focus here is on latent competencies, the appropriate model is then a hidden Markov model (HMM; e.g., Visser et al., 2002; Visser, 2011), and specifically an input-output HMM (Bengio and Frasconi, 1995). A HMM would allow us to infer about the efficacy of the learning supports in making a change in the *latent* state (proficiency level). In addition, the input-output HMM will allow us to make the association between learning materials (as input) and the change in KSA (latent) based on the observables (output), to estimate the contribution (efficacy) of each particular support to the desired change in proficiency (i.e., learning). Figure 7 illustrates this model for a single latent skill (KSA at time t1 and t2), a single observation (O at time t1 and t2) and a single learning support (l at time t1 and t2). The observation dependency on the skill (i.e., O given KSA; the arrow/link from KSA to O) is modeled by the Observational-Evidence model (the model from the original ECD), while the skill dependency on the learning support (i.e., KSA given l; the arrow/link from l to KSA) is modeled by the Transitional-Evidence model.

Working with the above example, let us assume a student does not know how to identify a data trend from a graph, and thus cannot extrapolate a new data point (incorrectly answers a question that requires extrapolation). Suppose a task support is provided, such that it draws the student’s attention to the pattern and trend in the data. We now want to estimate the contribution of this support in helping the student learn (and compare this contribution to other task supports). We have the following observables: the student’s incorrect answer in the first attempt, the student’s use of the particular task support, and the student’s revised answer in the second attempt (whether correct or not). Using an input-output HMM will allow us to estimate the probability of transitioning from the incorrect to the correct latent state (or in other cases from low proficiency to high proficiency), given the use of the task support. Of course, the model will be applied across questions and students in order to infer about latent state.

The above example of a single latent skill can be extended to a map of interconnected skills using dynamic Bayesian network (DBN; Murphy and Russell, 2002). DBN generalizes HMM by allowing the state space to be represented in a factored form instead of as a single discrete variable. DBN extends Bayesian networks (BN) to deal with changing situations.

How do we link the learning materials (defined in the Task-support model) to the learning processes/goals (defined in the KSA-change model)? Similar to the Q-matrix in the Observational-Evidence model, here too we need a matrix that links the learning materials (task supports) with the associated skills-change. We can use an S-matrix (Chen et al., 2018), which is a matrix of <supports × skills> (supports in the rows; skills in the columns), defining for each support which skills/process it can improve. In that sense, and similar to the Q-matrix, an S-matrix is a collection of “evidence” that explicate the connection between the supports and the desired learning shifts. For example, *providing a worked example* is a learning support that may be connected to several knowledge shifts (corresponding to subskills in the learning models), and providing opportunities for practice is another learning support that may be connected to different desired knowledge shifts (corresponding to different subskills). The S-matrix will specify these connections. The S-matrix will then play a role in the HMM, to determine the probability that a particular knowledge shift (learning process) occurred given the particular learning supports. Similar to the Q-matrix, the S-matrix should be determined by content experts, and/or learned or updated from the data.

## The e-Assembly Model

In the original ECD, the Assembly model determines how to put it all together and specifies the conditions needed for obtaining the desired reliability and validity for the assessment. In other words, it determines the *structure* of the test, the number and the mix of the desired items/tasks. The Assembly model is directly derived from the Proficiency model, such that it ensures, for example, the appropriate representation of all skills in the map. Going back to the HERA example and the KSA-model in Figure 3, if we were to build an assessment with those target skills, we would have to ensure that we sample items/tasks for each of the skills and subskills specified on the map, and the Assembly model will specify how much of each.

For the expanded ECD, we do not create a parallel model to the Assembly model as we did for the three core models, because in a blended learning and assessment system we do not assemble the assessment separately and the learning separately. Rather, in the process of developing a system, after we specified the six core models of the e-ECD, we assemble it all together in what we call the e-Assembly model.

The role of e-Assembly model is to specify how to put it all together. It will include the specifications of number and mix of items/tasks, but it will also include how and when to present the learning support materials. This can be seen as determining how to switch between the “assessment” mode of the system and the “learning” mode of the system.

The e-Assembly model provides an opportunity to take into account additional pedagogical principles that are relevant to the *combination* of items and tasks, such as the importance of reducing cognitive load for learning; focusing on one skill at a time; gradual increased difficulty presentation; adaptive presentation of content, among others. Conditions to ensure the validity of the system may also specify pedagogical principles such as learning *via* real-world authentic tasks or learning by doing, as well as learner engagement factors, as relevant. Pedagogical Content Knowledge principles that include knowledge of student *misconceptions* regarding specific phenomena, if articulated as part of the KSA and KSA-change model, should be also considered here in selecting and designing tasks, such that the misconceptions are either accounted for or avoided so the KSAs can be validly addressed.

The e-Assembly model is also the place to take into account considerations from other relevant approaches, such as the learner-centered design approach (LCD; Soloway et al., 1994; Quintana et al., 2000), which argue that student engagement and constructivist theories of learning should be at the core of a *computerized* learning system. Adopting such an approach will affect the combination and/or navigation through the system. For example, the system may guide students to be more *active* in trying out options and *making choices* regarding their navigation in the system.

An important aspect of systems for learning and assessment is whether they are adaptive to student performance and in what way. This aspect within the e-Assembly model ties directly to the e-Evidence model. The statistical models in the Evidence model are also good candidates for determining the adaptive algorithm in adaptive assessments. For example, if a 2PL IRT model is used to estimate ability; this model can also be used to select the items in a Computer Adaptive Test (CAT), as is often done in large-scale standardized tests that are adaptive (e.g., the old version of the GRE). Similarly, if a Bayes-net is used to estimate the map of KSAs, then the selection of items or tasks can be done based on the Bayes-net estimates of skills. Similarly, we can use the DCM to identify weakness in a particular skill and thus determine the next item that targets that particular weakness. This is true for any other model, also including data-driven models, because the purpose of the models is to provide a valid way to estimate KSAs, and once this is done, adaptivity within the system can be determined accordingly.

The learning aspect of the system is motivated by the goal to maximize learners’ gain and thus needs a more comprehensive adaptivity, or what is often called “recommendation model.” A recommendation model does not only determine the next item to be presented but it also determines which instructional or training material to recommend or present to the learner. A good recommendation model makes full use of all available information about both the learner and the instructional materials to maximize the KSA gain for the learner. If we have a way to estimate (measure) the gain for the learner, we can feed this information to the recommendation engine to determine the adaptivity in the form of the next task support and/or training and instructional material needed. Thus, the additional layer of an evidence model for the learning materials (i.e., the statistical models for estimating the efficacy of the task supports) provides a good candidate model for the recommendation engine. Which materials were already used by the learner (which ones were chosen/preferred), which supports are found more effective for that particular learner, which skill is currently in focus and which supports are most effective for that particular skill (e.g., practice, explained example, video lecture, simulation demonstration, providing instructional material for a prior/prerequisite skill, etc.) are some of the decisions needed to be made by a recommendation engine, and these decisions rely on the statistical models that were used to evaluate and provide evidence for the efficacy of the task support and instructional materials.

## Conclusion and Future Steps

In this paper, we propose a new way to fuse learning and assessment at the design stage. Specifically, we propose an expanded framework we developed to aid with the creation of a system for blended assessment and learning. We chose the ECD framework as a starting point because this is a comprehensive and rigorous framework for the development of assessments and underlies the development of tests for most testing organizations. Incorporating learning aspects, both learning goals and learning processes, in the ECD framework is challenging, because of fundamental differences in the assumptions and approaches of learning and assessment. Nevertheless, we showed that the unique structure of Proficiency, Task, and Evidence models lends itself to creating parallel models for consideration of the corresponding aspect of learning within each model.

We are currently applying this framework in our work. In future work, we hope to show examples of the learning and assessment system that we build following the e-ECD framework. We are also working to incorporate other elements into the framework, primarily the consideration of motivation, meta-cognition, and other non-cognitive skills. Since learners’ engagement is a crucial element in a learning system, we can think of a way to incorporate elements that enhance engagement as part of the assembly of the system, by using reward system or gamification in the form of points, coins, badges, etc. Adding gamification or engagement-enhancing elements into a system does not currently have a designated model within the e-ECD. We are working to find a way to incorporate these elements into the framework.

## Author Contributions

MA-A and AAvD contributed to the conception of the framework. MA-A contributed to the conception and specifications of the new models, and AAvD contributed to the CP component. SW and JT contributed to the e-Task model. BD contributed to the e-Evidence model. The authors would like to thank the reviewers for substantial contribution.

### Conflict of Interest Statement

The authors declare that the research was conducted in the absence of any commercial or financial relationships that could be construed as a potential conflict of interest.
